# Virulence Determinants and Methicillin Resistance in Biofilm-Forming *Staphylococcus aureus* from Various Food Sources in Bangladesh

**DOI:** 10.3390/antibiotics11111666

**Published:** 2022-11-20

**Authors:** Fatimah Muhammad Ballah, Md. Saiful Islam, Md. Liton Rana, Md. Ashek Ullah, Farhana Binte Ferdous, Fahim Haque Neloy, Samina Ievy, Md. Abdus Sobur, AMM Taufiquer Rahman, Mst. Minara Khatun, Marzia Rahman, Md. Tanvir Rahman

**Affiliations:** 1 Department of Microbiology and Hygiene, Faculty of Veterinary Science, Bangladesh Agricultural University, Mymensingh 2202, Bangladesh; 2 Department of Veterinary Public Health and Preventive Medicine, Usmanu Danfodiyo University, Sokoto 840004, Nigeria; 3 Naogaon District Hospital, Naogaon 6500, Bangladesh

**Keywords:** *S. aureus*, toxin, biofilm, antibiotic resistance, MRSA, *mecA*, beta-lactams

## Abstract

The eradication of staphylococcal infections has become more difficult due to the development of antibiotic resistance and virulence in biofilm-forming *Staphylococcus aureus*. The presence of the life-threatening zoonotic pathogen, methicillin-resistant *S. aureus* (MRSA), in foods indicates a public health issue. This study, therefore, aimed to determine virulence factors and methicillin resistance in biofilm-forming *S. aureus* isolates from different foods and food handlers. A total of 100 PCR-positive *S. aureus* isolates (97 biofilm formers and three non-biofilm formers) were screened using the disk diffusion method and PCR assay. By PCR, genes encoding virulence factors, e.g., enterotoxin (*sea*, 30%, 95% CI: 21.90–39.59%), toxic shock syndrome toxin (*tst*, 20%, 95% CI: 13.34–28.88%), and Panton–Valentine leukocidin toxin (*PVL*, 15%, 95% CI: 9.31–23.28%), were detected in the *S. aureus* isolates. By the disk diffusion method, 100% (95% CI: 96.30–100.00%) of *S. aureus* isolates were phenotypically MRSA in nature, showing 100% resistance to oxacillin and cefoxitin. Moreover, the methicillin-resistant gene *mecA* was found in 61 (61%, 95% CI: 51.20–69.98%) MRSA isolates. Furthermore, all the *S. aureus* isolates were phenotypically resistant to ampicillin and penicillin, 30% to erythromycin, and 11% to gentamycin. Among them, 51% (95% CI: 41.35–60.58%) of *S. aureus* isolates were phenotypically multidrug-resistant in nature, and the multiple antibiotic resistance index varied from 0.33 to 0.55. Genes encoding resistance to beta-lactams (*blaZ*, 100%, 95% CI: 96.30–100.00%) and tetracyclines (*tetA* and *tetC*, 3%, 95% CI: 0.82–8.45%) were found positive in the *S. aureus* isolates. Genes encoding virulence determinants and MRSA were significantly (*p* < 0.05) higher in strong biofilm-forming *S. aureus* than in moderate and non-biofilm-forming isolates. To our knowledge, this is the first study in Bangladesh to incorporate preliminary data on the occurrence of virulence determinants and methicillin resistance, including resistance to clinically important antibiotics, in biofilm-forming *S. aureus* isolates from different foods and food handlers in Bangladesh, emphasizing a potential threat to human health.

## 1. Introduction

*Staphylococcus aureus*, an opportunistic and notorious zoonotic pathogen, is responsible for food poisoning and a wide range of infections in humans, ranging from skin infections, diarrhea, nausea, vomiting, and abdominal cramps to serious consequences, such as endocarditis, pneumonia, osteomyelitis, toxic shock syndrome, and septicemia [1,2]. The consumption of *S. aureus*-contaminated foods is a major factor in the development of staphylococcal food poisoning in humans.

The pathogenicity of *S. aureus* is triggered by a number of characteristics, such as invasive components, toxin-associated virulence factors, biofilm formation, and antibiotic resistance. These characteristics also assist these organisms in becoming more resistant to hostile environments, developing infections, and escaping the immune system of the host [3,4,5].

The ability of *S. aureus* to form biofilm can shield them from antibiotics, enzymes released from the host immune system, and environmental stressors [6]. The formation of biofilms, which consist of an aggregation of microbial cells encased in exopolymeric substances, is a frequent strategy that bacteria use in order to survive in a variety of hostile environmental conditions [7]. Bacteria permanently alter their growth rate and gene transcription through the process of biofilm development, in which they cling to and grow on a surface and secrete extracellular polymers that aid adhesion and matrix creation [8]. The biofilm-forming ability of bacterial communities assists them in being resilient against environmental stressors, antimicrobials, or sanitizers that a single bacterium cannot [4].

Staphylococcal virulence factors such as (a) enterotoxins (*sea*, *seb*, and others) are responsible for food poisoning and help organisms become resistant to heat treatment [5], (b) toxic shock syndrome toxin (*tst*)—an exotoxin that causes rapid onset of fever, shock syndrome, hypotension, and inflammation of the vascular system [9], and (c) the Panton–Valentine leukocidin (*PVL*)—a cytotoxin that causes necrosis on the skin, lysis of human neutrophils—while also increasing *S. aureus* adherence to the extracellular matrix [10].

The use of antibiotics for treating bacterial infections has been increasing gradually since their discovery in the early nineteenth century [11]. However, selective pressure resulting from the misuse and overuse of antibiotics has triggered the development of antimicrobial resistance (AMR) or multidrug resistance (MDR) in many bacterial pathogens [12]. AMR is a major threat to human health and economic expansion [13]. Based on a predictive statistical model, there were an estimated 4.95 million bacterial AMR-associated deaths in 2019, with 1.27 million deaths from bacterial AMR [14]. The adverse impacts of AMR are more severe in low- and middle-income countries, including Bangladesh [15]. In addition, health components are being jeopardized by the consequences of AMR [16].

Antibiotic resistance in *S. aureus* has increased dramatically over the years. Consequently, a high degree of resistance developed in *S. aureus*, particularly in methicillin-resistant *S. aureus* (MRSA) strains, is a critical threat to human health [17]. A US study previously reported that MRSA causes more human mortalities than AIDS [18]. In addition, the superbug MRSA develops resistance to most of the available antibiotics which are used to treat staphylococcal infections [19]. *S. aureus* becomes resistant to methicillin, e.g., MRSA, by acquiring the *mecA*, *mecB*, or *mecC* genes. In MRSA, these resistant genes are harbored in a mobile genetic element *mec* operon, namely the staphylococcal cassette chromosome *mec* (*SCCmec*) [20]. MRSA showed MDR properties by developing resistance to beta-lactams, aminoglycosides, macrolides, fluoroquinolones, chloramphenicol, and tetracyclines, which are frequently used to treat staphylococcal infections [21].

Both the hand surfaces of food handlers and the surfaces that come into contact with foods are important factors in the spread of *S. aureus* in foods and settings that contain foods. As a result, *S. aureus* has been found in a variety of foods on multiple occasions [22]. Furthermore, MRSA is being increasingly detected in different food products, such as ready-to-eat food, hand swabs from food handlers, chicken products, etc. [17,23]. Indeed, no data are available from Bangladesh on the prevalence of virulence factors and methicillin resistance in biofilm-forming *S. aureus* from food origins. Considering the current importance, we conducted the present study focusing on the detection of staphylococcal virulence factors and MRSA with other clinically important antibiotic resistance genes and to determine their association with staphylococcal biofilm formation.

## 2. Results

### 2.1. Prevalence of Virulence Factors in Biofilm-Forming S. aureus

Out of 100 *S. aureus* isolates, 35 (95% CI: 26.36–44.75%) harbored at least one virulence gene, where the *sea*, *tst*, and *PVL* were detected in 30% (95% CI: 21.90–39.59%), 20% (95% CI: 13.34–28.88%), and 15% (95% CI: 9.31–23.28%) isolates, respectively. In addition, these three genes were significantly (*p* < 0.05) higher in strong biofilm-forming *S. aureus* isolates (*sea*: 100%; *tst*: 70%; *PVL*: 55%) than in moderate (*sea*: 12.98%; *tst*: 7.79%; *PVL*: 5.19%) and non-biofilm-forming (0% for all virulence genes) *S. aureus* isolates. None of the *S. aureus* isolates were found to be positive for the virulence *seb* gene. Moreover, the virulence genes *sea*, *tst*, and *PVL* were detected only in biofilm-forming *S. aureus* isolates, and non-biofilm-forming isolates did not harbor any virulence genes. Table 1 depicts the overall prevalence of different virulence genes detected in *S. aureus* isolates.

Sample-wise, *S. aureus* isolates detected from ready-to-eat foods showed the highest occurrence of different virulence genes compared with other samples (Supplementary Table S1).

Bivariate analysis revealed strong and positive significant correlations between virulence genes *sea* and *tst* (Pearson correlation coefficient, ρ = 0.600), *sea* and *PVL* (ρ = 0.458), and *tst* and *PVL* (ρ = 0.420). Table 2 shows the overall correlation outcomes between the virulence genes of *S. aureus* isolates.

### 2.2. Antibiogram Profiles of Biofilm-Forming S. aureus

**Figure 1:**
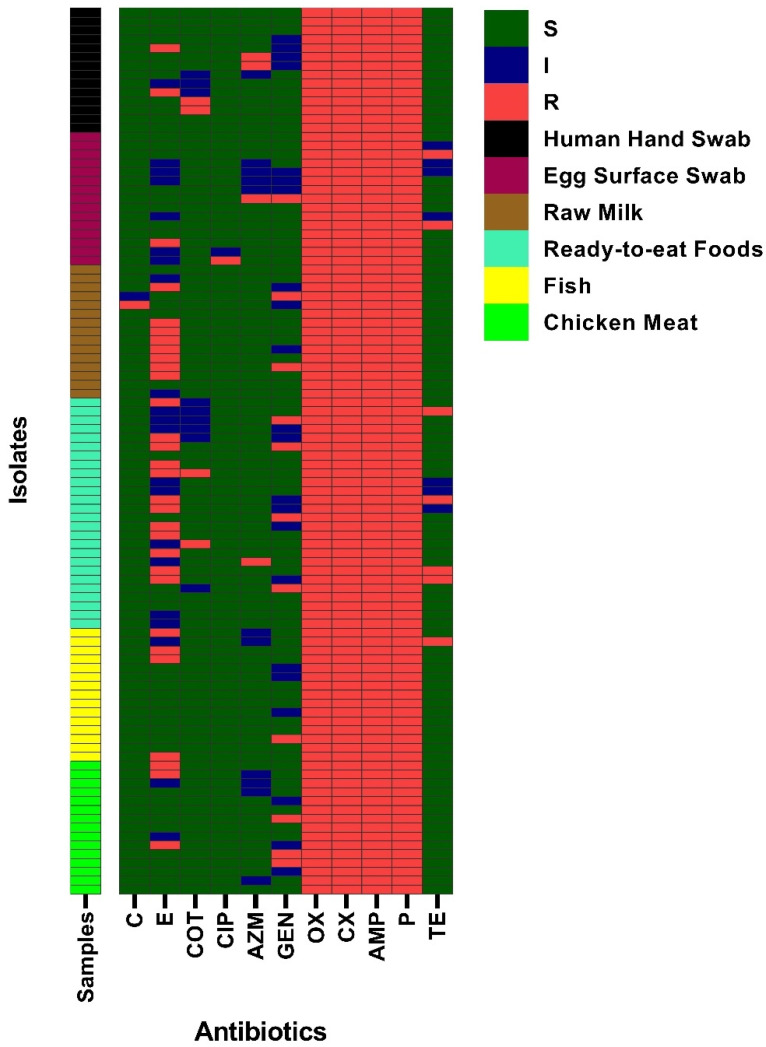
Antibiogram profiles of biofilm-forming *S. aureus* isolated from different foods and human hand swab samples. C = Chloramphenicol, E = Erythromycin, COT = Co-trimoxazole, CIP = Ciprofloxacin, AZM = Azithromycin, GEN = Gentamicin, OX = Oxacillin, AMP = Ampicillin, TE = Tetracycline, P = Penicillin, CX = Cefoxitin, S = Sensitive, I = Intermediate, and R = Resistant.

By disk diffusion assay, all the isolated *S. aureus* exhibited resistance to oxacillin and cefoxitin (100/100, 95% CI: 96.30–100%), which indicates that all the 100 isolates were phenotypically MRSA in nature. In addition, resistance to ampicillin and penicillin was found in every single isolate. Resistance to erythromycin and gentamicin was estimated at 30% and 11%, respectively. Sensitivity to chloramphenicol, ciprofloxacin, co-trimoxazole, tetracycline, and azithromycin was estimated at 98%, 98%, 87%, 86%, and 85%, respectively. Figure 1 shows the overall antibiotic susceptibility profiles of 100 *S. aureus* isolates. Sample-wise phenotypic antibiotic resistance patterns are documented in Supplementary Table S1 and Figure 1. Moreover, there was no statistically significant correlation between any of the two antibiotics’ resistance in *S. aureus* isolates (Supplementary Table S2).

### 2.3. Association of Antibiotic Resistance Patterns with Biofilm-Forming S. aureus

The resistance patterns of all the antibiotics except oxacillin, ampicillin, penicillin, and cefoxitin (which showed 100% resistance in all levels of biofilm-forming isolates) were higher in biofilm-forming *S. aureus* isolates than in non-biofilm-forming isolates. Moreover, the occurrence of erythromycin resistance patterns was significantly (*p* < 0.05) higher in strong biofilm-formers (55%), compared with intermediate (24.68%) and non-biofilm-forming (0%) isolates. Strong biofilm-forming *S. aureus* isolates showed higher resistance to other antibiotics (except chloramphenicol and ciprofloxacin) compared with moderate and non-biofilm-producing *S. aureus* isolates, but there were no significant variations (*p* > 0.05) (Table 3).

### 2.4. Phenotypic MDR and MAR Nature in Biofilm-Forming S. aureus

Of 100 *S. aureus* isolates, 51 (95% CI: 41.35–60.58%) were phenotypically MDR in nature. A total of 12 resistance patterns were audited, among them, 11 were MDR patterns. The MDR pattern number 5 (E, OX, AMP, P, CX) was observed in the highest number of MDR *S. aureus* isolates (24/51, 47.06%, 95% CI: 34.05–60.48%). The resistance pattern number 12 (OX, AMP, P, CX) was not phenotypically MDR in nature, though it was found in 49 (95% CI: 39.42–58.65%) *S. aureus* isolates. Seven isolates exhibited resistance against four classes of antibiotics (patterns 1, 2, 3, and 4), comprising six antibiotics. Moreover, all 100 *S. aureus* isolates showed more than 0.2 MAR indices (MAR index: 0.33–0.55). The MDR and MAR index profiles of *S. aureus* isolates are arranged in Table 4.

### 2.5. Genotypic Prevalence of MRSA and other Antibiotic Resistance in Biofilm-Forming S. aureus

By PCR, the methicillin resistance gene *mecA* was found to be positive in 61% (95% CI: 51.20–69.98%) of *S. aureus* isolates, which was significantly (*p* < 0.05) higher in strong biofilm-formers (80%) compared with moderate/intermediate (58.44%) and non-biofilm (0%) formers (Table 5). Sample-wise, all types of samples contained the methicillin resistance gene *mecA* (Supplementary Table S1).

Moreover, all the biofilm-forming *S. aureus* isolates were found positive for at least one antibiotic resistance gene (Supplementary Table S1). The tetracycline resistance genes *tetA* and *tetC* were detected in 3% (95% CI: 0.82–8.45%) of *S. aureus* isolates, and the beta-lactam gene *blaZ* was in 100% of *S. aureus* isolates. However, there was not a significant difference (*p* > 0.05) between the level of biofilm formation and the occurrence of these resistance genes. No isolates were found to be positive for *tetC* (Table 5). Sample-wise data on different antibiotic resistance genes are given in Supplementary Table S1.

## 3. Discussion

Foods, especially ready-to-eat foods, are becoming incredibly popular, with an increased number of restaurants and street vendors around the globe, most notably in Bangladesh. Other food sources such as milk, meat, fish, and eggs always have a high demand among all classes of people. However, foods contaminated with *S. aureus* have the potential to cause food poisoning, generating serious public health risks. In addition, biofilm formation in *S. aureus* is responsible for different persistent or chronic staphylococcal infections. In this study, we present the first-time detection of virulence determinants and methicillin resistance in biofilm-forming *S. aureus* isolated from different foods and human hand swab samples in Bangladesh. Islam et al. [22] conducted almost-similar types of research in Bangladesh. They reported virulence factors and antibiotic resistance only in *S. aureus* isolates from food sources, but they did not focus on biofilm. In addition, we showed variations in the occurrence of virulence and antibiotic resistance with different degrees of biofilm formation in *S. aureus* isolates, but they did not.

The pathogenic characteristics of *S. aureus* isolates provide vital details on the isolates’ ability to develop human and animal infections. In this study, 30% of *S. aureus* isolates harbored at least one virulence gene, demonstrating their potential pathogenic and toxic characteristics. However, no isolates tested positive for the *seb* gene. Previously, Islam et al. [24] also reported various virulence genes in *S. aureus* isolates from food samples in Bangladesh, detecting a higher prevalence (ours vs. theirs) for *seb* (0% vs. 11.4%) and *PVL* (15% vs. 71.4%) genes and a lower prevalence for *sea* (30% vs. 25.7%) and *tst* (20% vs. 17.1%) genes, compared with our present study. In other countries, multiple previous studies detected virulence genes harboring *S. aureus* isolates with different observations from food samples such as raw milk, meat, eggs, ready-to-eat foods, fish, food handlers, etc. Mashouf et al. [25] detected virulence genes, *sea* (25.5%) and *seb* (4%) in *S. aureus* isolated from animal-originated foods in Iran; Puah et al. [26] detected *sea* (5.8%), *seb* (1.9%), *tst* (9.6%), and *PVL* (9.6%) in ready-to-eat foods in Malaysia; Rong et al. [27] reported *sea* (22.7%), *seb* (10.1%), *tst* (2.5%), and *PVL* (50.4%) in aquatic foods in China; Yang et al. [28] reported *sea* (33.3%), *seb* (36.2%), *tst* (7.3%), and *PVL* (11.6%) in retail ready-to-eat foods in China; and Adame-Gómez et al. [29] detected *sea* (53.1%), *seb* (3.1%), and *tst* (9.3%) in food, humans, and animals in Mexico. The disparities in the detection rate of virulence factors in *S. aureus* isolates might be due to variations in geographical distributions, sample sizes and types, detection rate, biofilm-forming abilities, the hygienic condition of the sampling sites, sampling methodologies, and other factors.

Staphylococcal enterotoxins (*sea*, *seb*, and others) produced by *S. aureus* are directly associated with staphylococcal food poisoning [30]. The higher prevalence of the *sea* gene than the *seb* gene in *S. aureus* isolates detected in the present study is not unusual because the isolates with SEA-type toxins cause the most staphylococcal infections and outbreaks, followed by isolates with other staphylococcal enterotoxin-related infections [24]. Detecting genes encoding staphylococcal enterotoxins in the isolated staphylococcal species indicates a serious public health concern since these toxins have resistance activity against high temperatures and can even retain their biological properties in milk during pasteurization [31]. The *tst* gene is related mainly to human *S. aureus* isolates [24]. The presence of the *tst* gene in food and human hand swab samples suggests that this type of gene could be transferred from humans to animals and vice versa via the food chain. Another virulence gene, *PVL*, is a pertinent *S. aureus* virulence gene that is attributed mostly to community-acquired infections [32]. The detection of the *PVL* gene in our study suggests that food can be contaminated by this virulence gene, and these spoiled foods could perhaps constitute a source of community-acquired infections. In addition, virulence genes were found to be significantly higher in strong biofilm-forming *S. aureus* isolates. This indicates that as the degree of biofilm formation in *S. aureus* isolates increases, so does their ability to develop infections.

Treatment of staphylococcal infections relies mostly on antibiotic therapy; however, it frequently fails due to their resistance to antibiotics. In addition, the presence of MRSA in foods and on food handlers’ hands raises serious public health implications. In this study, all of the isolates were phenotypically MRSA in nature, indicating a critical threat to consumers by limiting the treatment options. Animal-originated food and food products can be contaminated with MRSA from infected animals, and processed food can be contaminated by infected vendors during food processing. The detection of MRSA in food and human hand swab samples indicates a serious issue for human health because it could be transmitted to humans via the food supply chain, causing staphylococcal infection. In addition, MRSA strains are responsible for severe morbidity and mortality in hospitals and even in healthy individuals [33]. Previously in Bangladesh, Islam et al. [24] reported that 25.7% (9/35) of *S. aureus* isolates originating from food samples were MRSA in nature, which is relatively lower than our findings. However, Saber et al. [17] recorded a similar prevalence rate, detecting MRSA in 100% of food samples in Egypt. In addition, previous studies found MRSA in different foods and human hand samples with lower and higher occurrence rates, i.e., 57.1% in food handlers in Brazil [34]; 10.1% in retail ready-to-eat foods [35] and 8.4% in retail aquatic products [27] in China; 12.94% in retail chicken meat and eggs in Nepal [36]; 42.3% in street-vended foods in India [37]; and 22.6% in ready-to-eat meat sandwiches in Egypt [38]. It is possible that different strategies for the distribution and usage of antibiotics in humans and animals account for the varying rates of MRSA detection in food samples. Moreover, *S. aureus* isolates detected in this study were highly resistant to ampicillin (100%), penicillin (100), and erythromycin (30%), but highly sensitive to chloramphenicol.

In total, 51% of the isolates detected in this study were MDR in nature, and the MAR index of the isolates varied from 0.33 to 0.55, demonstrating that antibiotics were haphazardly used at the source of the contamination. According to Krumperman [39], isolates with a MAR index of more than 0.2 were thought to have come from a high-risk source of contamination where antibiotics are commonly used. In Bangladesh, antibiotics are readily available in the markets, and there are no proper established policies for their distribution and usage. Therefore, people use antibiotics without any prescriptions and/or consultation with human physicians or veterinarians. The overuse and misuse of antimicrobial agents in both humans and animals might be responsible for the development of MDR *S. aureus* isolates in different food sources.

The association analysis between the degree of biofilm formation and phenotypic antibiotic resistance revealed that most antibiotics (except ampicillin, penicillin, oxacillin, and cefoxitin) showed resistance only to biofilm-forming *S. aureus* isolates. Antibiotic resistance increased with the level of biofilm formation, including the determination of significantly higher resistance to erythromycin in strong biofilm-forming *S. aureus* isolates. Antimicrobial resistance in *S. aureus* has been reported to increase to 1000 times that of planktonic cells in the presence of biofilm [40]. Various factors explaining the unprecedented resistance of biofilm-forming *S. aureus* and other bacteria to antibiotics include: (1) lower or decreased metabolic and growth rates of biofilm-formers, which may render them intrinsically less sensitive to antibiotics; (2) the structure of the biofilm-EPS (extracellular polymeric substances) matrix that assists biofilm cells to reduce the access of antibiotics to regions of the biofilm; and (3) the distinct physiological characteristics of biofilm cells that help to express MDR efflux pumps and stress-response regulons for developing antibiotic resistance [41].

In our study, 61% (61/100) of biofilm-forming *S. aureus* isolates harbored the methicillin resistance gene, *mecA*. The standard gold method for detecting MRSA isolates is *mecA* detection via PCR [42]; however, in our study, the *mecA* gene was absent in 39% of *S. aureus* isolates that were phenotypically MRSA. The inconsistency of the correlation between phenotypic and genotypic resistance of MRSA might be due to the mutation of genes that result in non-functional proteins and the dearth of gene expression [43]. In addition, the absence of the *mecA* gene in the MRSA isolates might be due to the detection methods we used, or those MRSA isolates could harbor other methicillin resistance genes, such as *mecB*, *mecC*, or *mecD* [42]. Our findings suggest the possible presence of other intrinsic and extrinsic factors having the ability to compete with the *mecA* gene for developing MRSA. Furthermore, the resistance gene *mecA* was significantly higher in strong biofilm-forming *S. aureus* isolates compared with moderate and non-biofilm producers. The genotypic detection of MRSA in biofilm-forming *S. aureus* isolates from foods suggests a serious threat to human health because these resistance gene-containing isolates could easily be transferred to humans via the food supply chain. In addition, it would be challenging to manage these organisms clinically because of their biofilm-forming activities.

Penicillin-resistant *S. aureus* isolates could be interpreted as beta-lactamase resistance. Detecting the *blaZ* gene using PCR is also necessary to determine the occurrence of beta-lactamase-producing isolates [37]. In this study, *blaZ* was found positive in all the *S. aureus* isolates, which is relatively higher than the findings (69.23%) from a previous study [37]. Tetracycline resistance genes *tetA* and *tetC* were also detected in biofilm-forming *S. aureus* isolates. The detection of antibiotic resistance genes in our *S. aureus* isolates from foods and human hand swab samples suggests that these resistance genes might be transferred to other bacteria via horizontal transmission. We found these resistance genes in both foods and handlers’ hand swab samples, indicating a high-risk *S. aureus* contamination developed by the poor hygienic condition of the sampling sites.

## 4. Materials and Methods

### 4.1. Selection of S. aureus Isolates

*Staphylococcus aureus* (*n* = 100, biofilm producers = 97, and non-producers = 3) strains and their data were obtained from our previous study on detecting biofilm-producing *S. aureus* from different foods (raw milk, egg surface, chicken muscle, fish, and ready-to-eat foods) and humans’ hand swab samples [44]. Originally, *S. aureus* isolates were identified by culturing on mannitol salt (MS) agar plates, applying different bacteriological analytical methods (Gram’s staining, glucose, and mannitol utilization tests, coagulase test, catalase test, and Voges–Proskauer tests), and finally, employing the polymerase chain reaction (PCR) test targeting the *nuc* gene [44]. The biofilm-producing ability of *S. aureus* isolates was evaluated by qualitative (Congo red agar plate test), quantitative (crystal violet microtiter plate test), and genotypic (PCR) assays [44]. All the data related to biofilm-forming *S. aureus* are documented in Supplementary Table S1.

### 4.2. Molecular Detection of Virulence Factors

PCR-confirmed *S. aureus* isolates were subjected to a simplex PCR for the detection of virulence factors, namely staphylococcal enterotoxin A (*sea*), staphylococcal enterotoxin B (*seb*), *tst*, and *PVL* (Table 6).

The DNA for PCR was extracted from pure cultures of *S. aureus* using the boiling technique [51,52]. The genomic DNA was amplified using a PCR thermal cycler (ASTEC, Fukuoka, Japan). The PCR mixture was prepared following the previous study [44], and the PCR conditions were set following the previous studies mentioned in Table 6. The PCR products that had been amplified were then run through a gel electrophoresis machine (Nippon Genetics, Tokyo, Japan) using 1.5% agarose (Invitrogen, Waltham, MA, USA). After completing the gel run, the products were stained using ethidium bromide (HiMedia, Maharashtra, India) and checked for their expected amplicon sizes using an ultraviolet trans-illuminator (Biometra, Göttingen, Germany). A 100-bp DNA ladder (Promega, Madison, WI, USA) was used to compare the sizes of the bands.

### 4.3. Antimicrobial Susceptibility Testing (AST)

The AST of isolated *S. aureus* was analyzed by the disk diffusion test [53] on Mueller–Hinton agar (HiMedia, India) plates. The concentration of grown *S. aureus* cultures was maintained by comparing it with 0.5 McFarland solution (HiMedia, Maharashtra, India). Eleven commercially available antibiotics (from seven antibiotic classes) were employed, including amphenicols (chloramphenicol-30 μg), macrolides (azithromycin-30 μg and erythromycin-15 μg), sulfonamides (co-trimoxazole-25 μg), fluoroquinolones (ciprofloxacin-5 μg), aminoglycosides (gentamicin-10 μg), penicillins (oxacillin-10 μg, penicillin-10 μg, and ampicillin-25 μg), tetracyclines (tetracycline-30 μg), and cephalosporins (cefoxitin-30 μg). The results of the AST of *S. aureus* isolates were interpreted by the CLSI guidelines [54]. Isolates showing resistance to methicillin and cefoxitin were considered phenotypically MRSA in nature. All the antibiotic disks were collected from two different manufacturers (HiMedia, Maharashtra, India, and Oxoid, Hampshire, UK), and all the susceptibility tests were done three times to confirm the exact results. MDR isolates were those that showed resistance to more than two antimicrobial categories [55]. Moreover, the index of multiple antibiotic resistance (MAR) was enumerated by the previously described formula [39]:

$$\mathrm{MAR}\;\mathrm{index}=\frac{\mathrm{The}\;\mathrm{number}\;\mathrm{of}\;\mathrm{antimicrobials}\;\mathrm{to}\;\mathrm{which}\;a\;\mathrm{given}\;S.\;aureus\;\mathrm{strain}\;\mathrm{is}\;\mathrm{resistant}\;}{\mathrm{The}\;\mathrm{sum}\;\mathrm{of}\;\mathrm{antibiotics}\;\mathrm{to}\;\mathrm{which}\;\mathrm{an}\;\mathrm{isolate}\;\mathrm{was}\;\mathrm{subjected}}$$

### 4.4. Molecular Detection of MRSA with other Antibiotic Resistance Genes

All the *S. aureus* isolates were subjected to PCR to detect their genotypic antibiotic resistance profiles. The molecular detection of MRSA was performed by detecting the methicillin resistance gene (*mecA*) using a PCR assay. Moreover, the genes associated with resistance to beta-lactams (*blaZ*) and tetracyclines (*tetA*, *tetB*, and *tetC*) were also tested using PCR (Table 6). The same amplification method was used to detect antibiotic resistance genes in *S. aureus* isolates as was used to detect virulence genes.

### 4.5. Statistical Analysis

Excel 365 (Microsoft/Office 365, Redmond, WA, USA) was used to incorporate data; GraphPad Prism (Prism 8.4.2, San Diego, CA, USA) and the Statistical Package for Social Science (IBM SPSS 25.0, Chicago, IL, USA) were used to analyze data.

#### 4.5.1. Descriptive Analysis

Descriptive analysis was employed to calculate the prevalence of different variables. To estimate the prevalence, a binomial 95% confidence interval (CI) was enumerated by GraphPad Prism using a previous method [56]. Moreover, using SPSS, the variations in the occurrence of virulence and antibiotic resistance with the occurrence of different degrees of biofilm formation in *S. aureus* isolates were determined by the chi-square test for relatedness (with the Z-test for proportion) with a ≤ 0.05 significance *p*-value.

#### 4.5.2. Bivariate Analysis

Using SPSS, the Pearson correlation coefficients were calculated to check whether any of the two antibiotics resistant (phenotypically) to *S. aureus* were correlated (statistically significant). Only those antibiotics that did not show constant resistance against *S. aureus* isolates were analyzed for the bivariate analysis. Moreover, a correlation between any two virulence genes was also determined. The significance level for the correlation was selected at *p* ≤ 0.05.

## 5. Conclusions

As stated, our study detected virulence determinants and methicillin resistance in biofilm-forming *S. aureus* isolates sourced from different foods and human hand swab samples for the first time in Bangladesh. This study shows a high prevalence rate of genes encoding virulence factors and methicillin resistance in *S. aureus* isolates. The *S. aureus* isolates also showed resistance to several clinically important antibiotics, which demonstrates a potential public health concern by transferring to humans from foods via the food supply chain. In addition, as revealed in the present study, their presence in foods and food handlers indicates that foods such as raw milk, chicken meat, ready-to-eat foods, fish, and eggs could be a possible source of virulent MRSA with significant clinical relevance. Standardized surveillance and monitoring programs, combined with an organized educational awareness campaign on AMR and good hygiene practices, should be implemented throughout the food production and supply chain to reduce the colonization and dissemination of virulent MRSA and biofilm strains and to guarantee the safety of foods.

## Figures and Tables

**Table 1:** Detection of virulence genes in biofilm-forming *S. aureus* strains (n = 100) isolated from different foods and hand swab samples.

Virulence Genes	Virulence in Different Degrees of Biofilm Formation			Total No. of Positive Isolates (%, 95% CI)	*p*-Value
	No. of Strong Biofilm Formers (n = 20)	No. of Intermediate Biofilm Formers (n = 77)	No. of Non-Biofilm Formers (n = 3)		
*sea*	20 (100% ^a^)	10 (12.98% ^b^)	0 (0% ^b^)	30 (30, 21.90–39.59%)	<0.001
*seb*	0 (0% ^a^)	0 (0% ^a^)	0 (0% ^a^)	0 (0, 00.00–3.70%)	NA
*tst*	14 (70% ^a^)	6 (7.79% ^b^)	0 (0% ^b^)	20 (20, 13.34–28.88%)	<0.001
*PVL*	11 (55% ^a^)	4 (5.19% ^b^)	0 (0% ^b^)	15 (15, 9.31–23.28%)	<0.001

Values with different superscripts differ significantly (*p* < 0.05) within the variable under assessment, CI = Confidence interval, NA = Not applied, n = number of isolates.

**Table 2:** Pearson correlation coefficient to assess the pairs of any of two virulence genes detected in biofilm-forming *S. aureus* isolated from different foods and human hand swab samples.

		*sea*	*seb*	*tst*	*PVL*
*sea*	ρ	1			
	*p*-value	-			
*seb*	ρ	.a	.a		
	*p*-value	-	-		
*tst*	ρ	0.600 **	.a	1	
	*p*-value	0.000	-	-	
*PVL*	ρ	0.458 **	.a	0.420 **	1
	*p*-value	0.000	-	0.000	-

ρ = Pearson correlation coefficient, ** Significant correlation at the 0.01 level (*p*-value), .a = Not calculable, as at least one of the input variables is fixed.

**Table 3:** Association of antibiotic resistance patterns and biofilm formation in *S. aureus* strains detected in different food and hand swab samples.

Antibiotics	Antibiotic Resistance in Different Degrees of Staphylococcal Biofilm Formation			Total No. of Resistant Isolates (%, 95% CI)	*p*-Value
	No. of Strong Biofilm Formers (n = 20)	No. of Intermediate Biofilm Formers (n = 77)	No. of Non-Biofilm Formers (n = 3)		
C	0 (0% ^a^)	1 (1.30% ^a^)	0 (0% ^a^)	1 (1, 0.05–5.45%)	0.860
E	11 (55% ^a^)	19 (24.68% ^b^)	0 (0% ^b^)	30 (30, 21.90–39.59%)	0.016
COT	1 (5% ^a^)	3 (3.90% ^a^)	0 (0% ^a^)	4 (4, 1.57–9.84%)	0.914
CIP	0 (0% ^a^)	1 (1.30% ^a^)	0 (0% ^a^)	1 (1, 0.05–5.45%)	0.860
AZM	2 (10% ^a^)	2 (2.60% ^a^)	0 (0% ^a^)	4 (4, 1.57–9.84%)	0.302
GEN	3 (15% ^a^)	8 (10.39% ^a^)	0 (0% ^a^)	11 (11, 6.25–18.63%)	0.695
OX	20 (100% ^a^)	77 (100% ^a^)	3 (100% ^a^)	100 (100, 96.30–100%)	NA
AMP	20 (100% ^a^)	77 (100% ^a^)	3 (100% ^a^)	100 (100, 96.30–100%)	NA
TE	3 (15% ^a^)	4 (5.20% ^a^)	0 (0% ^a^)	7 (7, 3.43–13.75%)	0.276
CX	20 (100% ^a^)	77 (100% ^a^)	3 (100% ^a^)	100 (100, 96.30–100%)	NA
P	20 (100% ^a^)	77 (100% ^a^)	3 (100% ^a^)	100 (100, 96.30–100%)	NA

Values with different superscripts differ significantly (*p* < 0.05) within the variable under assessment, CI = Confidence interval, NA= Not applied, n = number of isolates, C = Chloramphenicol, E = Erythromycin, COT = Co-trimoxazole, CIP = Ciprofloxacin, AZM = Azithromycin, GEN = Gentamicin, OX = Oxacillin, AMP = Ampicillin, TE = Tetracycline, P = Penicillin, and CX = Cefoxitin.

**Table 4:** MDR and MAR profiles of *S. aureus* isolates detected from different foods and hand swab samples.

No. of Pattern	Antibiotic Resistance Patterns	No. of Antibiotics (Classes)	No. of Isolates	Overall MDR Isolates (%)	MAR Index
1	E, COT, OX, AMP, P, CX	6 (4)	1	51/100 (51)	0.55
2	AZM, GEN, OX, AMP, P, CX	6 (4)	1		
3	E, OX, AMP, P, CX, TE	6 (4)	3		
4	E, GEN, OX, AMP, P, CX	6 (4)	2		
5	E, OX, AMP, P, CX	5 (3)	24		0.46
6	AZM, OX, AMP, P, CX	5 (3)	3		
7	GEN, OX, AMP, P, CX	5 (3)	8		
8	COT, OX, AMP, P, CX	5 (3)	3		
9	OX, AMP, P, CX, TE	5 (3)	4		
10	CIP, OX, AMP, P, CX	5 (3)	1		
11	C, OX, AMP, P, CX	5 (3)	1		
12 *	OX, AMP, P, CX	4 (2)	49	-	0.33

MDR = multidrug resistant, MAR = multiple antibiotic resistance, C = Chloramphenicol, E = Erythromycin, COT = Co-trimoxazole, CIP = Ciprofloxacin, AZM= Azithromycin, GEN = Gentamicin, OX = Oxacillin, AMP = Ampicillin, TE = Tetracycline, P = Penicillin, and CX = Cefoxitin; * Non-multidrug-resistant.

**Table 5:** Detection of antibiotic resistance genes in biofilm-forming *S. aureus* strains (n= 100) detected from different foods and hand swab samples.

Antibiotic Resistance Genes	Occurrence of Antibiotic Resistance Genes in Different Degrees of Staphylococcal Biofilm Formation			Total No. of Positive Isolates (%, 95% CI)	*p*-Value
	No. of Strong Biofilm Formers (n = 20)	No. of Intermediate Biofilm Formers (n = 77)	No. of Non-Biofilm Formers (n = 3)		
*mecA*	16 (80% ^a^)	45 (58.44% ^b^)	0 (0% ^b^)	61 (61, 51.20–69.98%)	0.0189
*blaZ*	20 (100% ^a^)	77 (100% ^a^)	3 (100% ^a^)	100 (100, 96.30–100.00%)	NA
*tetA*	0 (0% ^a^)	3 (3.90% ^a^)	0 (0% ^a^)	3 (3, 0.82–8.45%)	0.6301
*tetB*	0 (0% ^a^)	0 (0% ^a^)	0 (0% ^a^)	0 (0, 0.00–3.70%)	NA
*tetC*	2 (10% ^a^)	1 (1.30% ^a^)	0 (0% ^a^)	3 (3, 0.82–8.45%)	0.1209

Values with different superscripts differ significantly (*p* < 0.05) within the variable under assessment, CI = Confidence interval, NA = Not applied, n = number of isolates.

**Table 6:** Primers’ list associated with the virulence and antibiotic resistance.

Factors	Targeted Genes	Primer Sequence (5′–3′)	Annealing Temperature	Amplicon Size (Bp)	References
Antibiotic resistance	*mecA*	F: AAAATCGATGGTAAAGGTTGG R: AGTTCTGGCACTACCGGATTTTGC	55	533	[45]
	*tetA*	F: GGTTCACTCGAACGACGTCA R: CTGTCCGACAAGTTGCATGA	57	577	[46]
	*tetB*	F: CCTCAGCTTCTCAACGCGTG R: GCACCTTGCTCATGACTCTT	56	634	
	*tetC*	F: CTT GAGAGCCTTCAACCCAG R: ATG GTCGTCATCTACCTGCC	57	418	[47]
	*blaZ*	F: TCAAACAGTTCACATGCC R: TTCATTACACTCTGGCG	46	900	[48]
Virulence	*sea*	F: CCTTTGGAAACGGTTAAAACG R: TCTGAACCTTCCCATCAAAAAC	55	128	[49]
	*seb*	F: TCGCATCAAACTGACAAACG R: GCAGGTACTCTATAAGTGCCTGC	55	477	
	*tst*	F: AAGCCCTTTGTTGCTTGCG R: ATCGAACTTTGGCCCATACTTT	55	445	
	*PVL*	F: ATCATTAGGTAAAATGTCTGGACATGATCCA R: GCATCAAGTGTATTGGATAGCAAAAGC	55	433	[50]

## Data Availability

Not applicable.

## Supplementary Materials

The following supporting information can be downloaded at: https://www.mdpi.com/article/10.3390/antibiotics11111666/s1, Table S1: Distribution of genes associated with biofilm formation, antibiotic resistance patterns, and virulence profiles in *S. aureus* isolates; Table S2: Pearson correlation coefficients between any of two antibiotics showing resistance to *S. aureus* isolates detected from different foods and hand swab samples.

## References

[1] Rahman M., Sobur M., Islam M., Ievy S., Hossain M., El Zowalaty M.E., Rahman A.M.M., Ashour H.M. (2020). Zoonotic diseases: Etiology, impact, and control. Microorganisms.

[2] Urmi M.R., Ansari W.K., Islam M.S., Sobur M.A., Rahman M., Rahman M.T. (2021). Antibiotic resistance patterns of *Staphylococcus* spp. Isolated from fast foods sold in different restaurants of Mymensingh. Bangladesh. J. Adv. Vet. Anim. Res..

[3] Argudín M.Á., Mendoza M.C., Rodicio M.R. (2010). Food poisoning and *Staphylococcus aureus* enterotoxins. Toxins.

[4] Oliveira D., Borges A., Simões M. (2018). *Staphylococcus aureus* toxins and their molecular activity in infectious diseases. Toxins.

[5] Jain V.K., Singh M., Joshi V.G., Chhabra R., Singh K., Rana Y.S. (2022). Virulence and antimicrobial resistance gene profiles of *Staphylococcus aureus* associated with clinical mastitis in cattle. PLoS ONE.

[6] Ricciardi B.F., Muthukrishnan G., Masters E., Ninomiya M., Lee C.C., Schwarz E.M. (2018). *Staphylococcus aureus* evasion of host immunity in the setting of Prosthetic Joint Infection: Biofilm and beyond. Curr. Rev. Musculoskelet. Med..

[7] Costa O.Y., Raaijmakers J.M., Kuramae E.E. (2018). Microbial extracellular polymeric substances: Ecological function and impact on soil aggregation. Front. Microbiol..

[8] Donlan R.M. (2001). Biofilm Formation: A Clinically Relevant Microbiological Process. Clin. Infect. Dis..

[9] Poudel B., Zhang Q., Trongtorsak A., Pyakuryal B., Egoryan G., Sous M., Ahmed R., Trelles-Garcia D.P., Yanez-Bello M.A., Trelles-Garcia V.P. (2021). An overlooked cause of septic shock: Staphylococcal Toxic Shock Syndrome secondary to an axillary abscess. IDCases.

[10] Schaumburg F., Ngoa U.A., Kösters K., Köck R., Adegnika A.A., Kremsner P.G., Lell B., Peters G., Mellmann A., Becker K. (2011). Virulence factors and genotypes of *Staphylococcus aureus* from infection and carriage in Gabon. Clin. Microbiol. Infect..

[11] Islam M.S., Paul A., Talukder M., Roy K., Sobur M.A., Ievy S., Nayeem M.M.H., Rahman S., Nazir K.N.H., Hossain M.T. (2021). Migratory birds travelling to Bangladesh are potential carriers of multi-drug resistant *Enterococcus* spp., *Salmonella* spp., and *Vibrio* spp.. Saudi J. Bio. Sci..

[12] Islam M., Sobur M., Rahman S., Ballah F.M., Ievy S., Siddique M.P., Rahman M., Kafi M., Rahman M. (2022). Detection of bla_TEM_, bla_CTXM-M_, bla_CMY_, and bla_SHV_ Genes Among Extended-Spectrum Beta-Lactamase-Producing *Escherichia coli* Isolated from Migratory Birds Travelling to Bangladesh. Microb. Ecol..

[13] Ievy S., Islam M., Sobur M., Talukder M., Rahman M., Khan M.F.R. (2020). Molecular detection of avian pathogenic *Escherichia coli* (APEC) for the first time in layer farms in Bangladesh and their antibiotic resistance patterns. Microorganisms.

[14] Murray C.J., Ikuta K.S., Sharara F., Swetschinski L., Aguilar G.R., Gray A., Han C., Bisignano C., Rao P., Wool E. (2022). Global burden of bacterial antimicrobial resistance in 2019: A systematic analysis. Lancet.

[15] Roy K., Islam M.S., Paul A., Ievy S., Talukder M., Sobur M.A., Ballah F.M., Khan M.S.R., Rahman M.T. (2022). Molecular detection and antibiotyping of multi-drug resistant *Enterococcus faecium* from healthy broiler chickens in Bangladesh. Vet. Med. Sci..

[16] Talukder M., Islam M.S., Ievy S., Sobur M.A., Ballah F.M., Najibullah M., Rahman M.B., Rahman M.T., Khan M.F.R. (2021). Detection of multidrug resistant *Salmonella* spp. From healthy and diseased broilers having potential public health significance. J. Adv. Biotechnol. Exp. Ther..

[17] Saber T., Samir M., El-Mekkawy R.M., Ariny E., El-Sayed S.R., Enan G., Abdelatif S.H., Askora A., Merwad A.M., Tartor Y.H. (2021). Methicillin-and vancomycin-resistant *Staphylococcus aureus* from humans and ready-to-eat meat: Characterization of antimicrobial resistance and biofilm formation ability. Front. Microbiol..

[18] Klevens R.M., Morrison M.A., Nadle J., Petit S., Gershman K., Ray S., Harrison L.H., Lynfield R., Dumyati G., Townes J.M. (2007). Invasive methicillin-resistant *Staphylococcus aureus* infections in the United States. JAMA.

[19] Hossain M.J., Islam M.S., Sobur M.A., Zaman S.B., Nahar A., Rahman M., Rahman M.T. (2020). Exploring poultry farm environment for antibiotic resistant *Escherichia coli*, *Salmonella* spp., and *Staphylococcus* spp. having public health significance. J. Bangladesh Agric. Univ..

[20] Rahman M.T., Kobayashi N., Alam M.M., Ishino M. (2005). Genetic analysis of *mecA* homologues in *Staphylococcus sciuri* strains derived from mastitis in dairy cattle. Microb. Drug Resist..

[21] Piechota M., Kot B., Frankowska-Maciejewska A., Gruzewska A., Woźniak-Kosek A. (2018). Biofilm formation by Methicillin-resistant and Methicillin-sensitive *Staphylococcus aureus* strains from hospitalized patients in Poland. BioMed Res. Int..

[22] Chen Q., Xie S., Lou X., Cheng S., Liu X., Zheng W., Zheng Z., Wang H. (2020). Biofilm formation and prevalence of adhesion genes among *Staphylococcus aureus* isolates from different food sources. Microbiologyopen.

[23] Li H., Andersen P.S., Stegger M., Sieber R.N., Ingmer H., Staubrand N., Dalsgaard A., Leisner J.J. (2019). Antimicrobial Resistance and Virulence Gene Profiles of Methicillin-Resistant and -Susceptible *Staphylococcus aureus* from Food Products in Denmark. Front. Microbiol..

[24] Islam M.A., Parveen S., Rahman M., Huq M., Nabi A., Khan Z.U.M., Ahmed N., Wagenaar J.A. (2019). Occurrence and characterization of methicillin resistant *Staphylococcus aureus* in processed raw foods and ready-to-eat foods in an urban setting of a developing country. Front. Microbiol..

[25] Mashouf R.Y., Hosseini S.M., Mousavi S.M., Arabestani M.R. (2015). Prevalence of enterotoxin genes and antibacterial susceptibility pattern of *Staphylococcus aureus* strains isolated from animal originated foods in West of Iran. Oman Med. J..

[26] Puah S.M., Chua K.H., Tan J.A.M.A. (2016). Virulence Factors and Antibiotic Susceptibility of *Staphylococcus aureus* Isolates in Ready-to-Eat Foods: Detection of *S. aureus* Contamination and a High Prevalence of Virulence Genes. Int. J. Environ. Res. Public Health.

[27] Rong D., Wu Q., Xu M., Zhang J., Yu S. (2017). Prevalence, virulence genes, antimicrobial susceptibility, and genetic diversity of *Staphylococcus aureus* from retail aquatic products in China. Front. Microbiol..

[28] Yang X., Yu S., Wu Q., Zhang J., Wu S., Rong D. (2018). Multilocus Sequence Typing and Virulence-Associated Gene Profile Analysis of *Staphylococcus aureus* Isolates from Retail Ready-to-Eat Food in China. Front. Microbiol..

[29] Adame-Gómez R., Castro-Alarcón N., Vences-Velázquez A., Toribio-Jiménez J., Pérez-Valdespino A., Leyva-Vázquez M.A., Ramírez-Peralta A. (2020). Genetic diversity and virulence factors of S. aureus isolated from food, humans, and animals. Int. J. Microbiol..

[30] Lv G., Jiang R., Zhang H., Wang L., Li L., Gao W., Zhang H., Pei Y., Wei X., Dong H. (2021). Molecular Characteristics of *Staphylococcus aureus* From Food Samples and Food Poisoning Outbreaks in Shijiazhuang, China. Front. Microbiol..

[31] Zschöck M., Kloppert B., Wolter W., Hamann H.P., Lämmler C.H. (2005). Pattern of enterotoxin genes *seg*, *seh*, *sei* and *sej* positive *Staphylococcus aureus* isolated from bovine mastitis. Vet. Microbiol..

[32] Wang X., Li G., Xia X., Yang B., Xi M., Meng J. (2014). Antimicrobial susceptibility and molecular typing of methicillin-resistant *Staphylococcus aureus* in retail foods in Shaanxi, China. Foodborne Pathog. Dis..

[33] Weber J.T. (2005). Community-associated methicillin-resistant *Staphylococcus aureus*. Clin. Infect. Dis..

[34] Ferreira J.S., Costa W.L.R., Cerqueira E.S., Carvalho J.S., Oliveira L.C., Almeida R.C.C. (2014). Food handler-associated methicillin-resistant *Staphylococcus aureus* in public hospitals in Salvador, Brazil. Food Control.

[35] Yang X., Zhang J., Yu S., Wu Q., Guo W., Huang J., Cai S. (2016). Prevalence of *Staphylococcus aureus* and methicillin-resistant *Staphylococcus aureus* in retail ready-to-eat foods in China. Front. Microbiol..

[36] Devkota S.P., Paudel A., Gurung K. (2019). Vancomycin Intermediate MRSA Isolates Obtained from Retail Chicken Meat and Eggs Collected at Pokhara, Nepal. Nepal J. Biotechnol..

[37] Sivakumar M., Dubal Z.B., Kumar A., Bhilegaonkar K., Vinodh Kumar O.R., Kumar S., Kadwalia A., Shagufta B., Grace M.R., Ramees T.P. (2019). Virulent methicillin resistant *Staphylococcus aureus* (MRSA) in street vended foods. J. Food Sci. Technol..

[38] Mahros M.A., Abd-Elghany S.M., Sallam K.I. (2021). Multidrug-, methicillin-, and vancomycin-resistant *Staphylococcus aureus* isolated from ready-to-eat meat sandwiches: An ongoing food and public health concern. Int. J. Food Microbiol..

[39] Krumperman P.H. (1983). Multiple antibiotic resistance indexing of *Escherichia coli* to identify high-risk sources of fecal contamination of foods. Appl. Environ. Microbiol..

[40] Guo Y., Song G., Sun M., Wang J., Wang Y. (2020). Prevalence and therapies of antibiotic-resistance in *Staphylococcus aureus*. Front. Cell. Infect. Microbiol..

[41] Davies D. (2003). Understanding biofilm resistance to antibacterial agents. Nat. Rev. Drug Discov..

[42] Sobur M., Islam M., Haque Z.F., Orubu E.S.F., Toniolo A., Choudhury M., Rahman M. (2022). Higher seasonal temperature enhances the occurrence of methicillin resistance of Staphylococcus aureus in house flies (*Musca domestica*) under hospital and environmental settings. Folia Microbiol..

[43] Martineau F., Picard F.J., Lansac N., Ménard C., Roy P.H., Ouellette M., Bergeron M.G. (2000). Correlation between the resistance genotype determined by multiplex PCR assays and the antibiotic susceptibility patterns of *Staphylococcus aureus* and *Staphylococcus epidermidis*. Antimicrob. Agents Chemother..

[44] Ballah F.M., Islam M.S., Rana M.L., Ferdous F.B., Ahmed R., Pramanik P.K., Karmoker J., Ievy S., Sobur M.A., Siddique M.P. (2022). Phenotypic and Genotypic Detection of Biofilm-Forming *Staphylococcus aureus* from Different Food Sources in Bangladesh. Biology.

[45] Lee J.H. (2003). Methicillin (oxacillin)-resistant *Staphylococcus aureus* strains isolated from major food animals and their potential transmission to humans. Appl. Environ. Microbiol..

[46] Randall L.P., Cooles S.W., Osborn M.K., Piddock L.J., Woodward M.J. (2004). Antibiotic resistance genes, integrons and multiple antibiotic resistance in thirty-five serotypes of *Salmonella enterica* isolated from humans and animals in the UK. J. Antimicrob. Chemother..

[47] Ng L.K., Martin I., Alfa M., Mulvey M. (2001). Multiplex PCR for the detection of tetracycline resistant genes. Mol. Cell. Probes.

[48] Rosato A.E., Kreiswirth B.N., Craig W.A., Eisner W., Climo M.W., Archer G.L. (2003). *mecA*-*blaZ* corepressors in clinical *Staphylococcus aureus* isolates. Antimicrob. Agents Chemother..

[49] Becker K., Roth R., Peters G. (1998). Rapid and Specific Detection of Toxigenic Staphylococcus aureus: Use of Two Multiplex PCR Enzyme Immunoassays for Amplification and Hybridization of Staphylococcal Enterotoxin Genes, Exfoliative Toxin Genes, and Toxic Shock Syndrome Toxin 1 Gene. J. Clin. Microbiol..

[50] Lina G., Piémont Y., Godail-Gamot F., Bes M., Peter M.O., Gauduchon V., Vandenesch F., Etienne J. (1999). Involvement of panton-valentine leukocidin—Producing *Staphylococcus aureus* in primary skin infections and pneumonia. Genet. Mol. Res..

[51] Islam M.S., Nayeem M.M.H., Sobur M.A., Ievy S., Islam M.A., Rahman S., Kafi M.A., Ashour H.M., Rahman M.T. (2021). Virulence determinants and multidrug resistance of *Escherichia coli* isolated from migratory birds. Antibiotics.

[52] Tawyabur M., Islam M., Sobur M., Hossain M., Mahmud M., Paul S., Hossain M.T., Ashour H.M., Rahman M. (2020). Isolation and characterization of multidrug-resistant *Escherichia coli* and *Salmonella* spp. from healthy and diseased turkeys. Antibiotics.

[53] Bayer A.W., Kirby W.M., Sherris J.C., Turck M. (1966). Antibiotic susceptibility testing by a standardized single disc method. Am. J. Clin. Pathol..

[54] CLSI (2020). Performance Standards for Antimicrobial Susceptibility Testing.

[55] Sweeney M.T., Lubbers B.V., Schwarz S., Watts J.L. (2018). Applying definitions for multidrug resistance, extensive drug resistance and pandrug resistance to clinically significant livestock and companion animal bacterial pathogens. J. Antimicrob. Chemother..

[56] Brown L.D., Cai T.T., DasGupta A. (2001). Interval estimation for a binomial proportion. Stat. Sci..

