# Supplementary material for: Virulence Determinants and Methicillin Resistance in Biofilm-Forming Staphylococcus aureus from Various Food Sources in Bangladesh
Source: Antibiotics (Basel). 2022 Nov 20;11(11):1666. doi: 10.3390/antibiotics11111666 (PMC9686753; doi:10.3390/antibiotics11111666)
Supplement: Supplementary file 1 [file antibiotics-11-01666-s001.zip › antibiotics-2038366-supplementary.pdf]

Supplementary Data

Virulence Determinants and Methicillin Resistance in Biofilm-Forming *Staphylococcus aureus* from Various Food Sources in Bangladesh

Fatimah Muhammad Ballah[1,2], Md. Saiful Islam[1], Md. Liton Rana[1], Md. Ashek Ullah[1], Farhana Binte Ferdous[1], Fahim Haque Neloy[1], Samina Ievy[1], Md. Abdus Sobur[1], AMM Taufiquer Rahman[3], Mst. Minara Khatun[1], Marzia Rahman[1], Md. Tanvir Rahman[1*]

[1]Department of Microbiology and Hygiene, Faculty of Veterinary Science, Bangladesh Agricultural University, Mymeningh-2202. Bangladesh

[2]Department of Veterinary Public Health and Preventive Medicine, Usmanu Danfodiyo University, Sokoto 840004, Nigeria

[3]Naogaon District Hospital. Naogaon- 6500, Bangladesh

*Corresponding author: tanvirahman@bau.edu.bd

Supplementary Table S1. Distribution of genes associated with biofilm formation, antibiotic resistance patterns, and virulence profiles in *S. aureus* isolates

SL No.	Source	Name of isolates	Degree of biofilm-formation	Biofilm-associated genes	Antibiotic resistance patterns		Virulence-associated genes
					Phenotypic	Genotypic	
1		HHS3	Strong	*icaA*, *icaB*, *icaC*, *icaD*	OX, AMP, P, CX	*mecA*, *blaZ*	*sea*, *tst*

2	Human hand swabs	HHS8	Intermediate	-	OX, AMP, P, CX	*mecA*, *blaZ*	-
3		HHS10	Intermediate	-	OX, AMP, P, CX	*mecA*, *blaZ*	-
4		HHS19	Intermediate	*icaB*, *icaD*, *bap*	OX, AMP, P, CX	*blaZ*	-
5		HHS6	Intermediate	-	E, OX, AMP, P, CX	*blaZ*	-
6		HHS2	Strong	*icaB*, *icaD*, *bap*	AZM, OX, AMP, P, CX	*blaZ*	*sea*, *tst*, *PVL*
7		HHS1	Intermediate	-	AZM, OX, AMP, P, CX	*blaZ*	-
8		HHS26	Intermediate	-	OX, AMP, P, CX	*mecA*, *blaZ*	-
9		HHS9	Intermediate	-	OX, AMP, P, CX	*blaZ*	*PVL*
10		HHS41	Intermediate	-	E, OX, AMP, P, CX	*mecA*, *blaZ*	-
11		HHS5	Intermediate	-	COT, OX, AMP, P, CX	*mecA*, *blaZ*	-
12		HHS4	Intermediate	-	COT, OX, AMP, P, CX	*mecA*, *blaZ*	-
13		HHS11	Non-former	-	OX, AMP, P, CX	*blaZ*	-
14		HHS32	Intermediate	-	OX, AMP, P, CX	*blaZ*	-
15	Egg surfaces	ES4	Intermediate	*icaB*, *icaD*	OX, AMP, P, CX	*mecA*, *blaZ*	*sea*
16		ES1	Intermediate	-	OX, AMP, P, CX	*blaZ*	-
17		ES15	Intermediate	-	OX, AMP, P, CX, TE	*mecA*, *tetA*, *blaZ*	-
18		ES22	Intermediate	-	OX, AMP, P, CX	*mecA*, *blaZ*	-
19		ES3	Intermediate	-	OX, AMP, P, CX	*mecA*, *blaZ*	*PVL*
20		ES12	Intermediate	*icaA*, *icaB*, *icaD*, *bap*	OX, AMP, P, CX	*blaZ*	-
21		ES51	Intermediate	-	OX, AMP, P, CX	*mecA*, *blaZ*	-
22		ES39	Intermediate	-	AZM, GEN, OX, AMP, P, CX	*mecA*, *blaZ*	*sea*

23		ES25	Intermediate	-	OX, AMP, P, CX	*mecA*, *blaZ*	-
24		ES16	Intermediate	-	OX, AMP, P, CX	*mecA*, *blaZ*	-
25		ES13	Intermediate	-	OX, AMP, P, CX, TE	*mecA*, *tetC*, *blaZ*	-
26		ES60	Intermediate	-	OX, AMP, P, CX	*blaZ*	-
27		ES48	Intermediate	-	E, OX, AMP, P, CX	*mecA*, *blaZ*	-
28		ES8	Intermediate	*icaA*, *icaB*, *icaD*, *bap*	OX, AMP, P, CX	*mecA*, *blaZ*	-
29		ES55	Intermediate	-	CIP, OX, AMP, P, CX	*mecA*, *blaZ*	-
30	Raw milk	RM6	Intermediate	-	OX, AMP, P, CX	*mecA*, *blaZ*	-
31		RM4	Intermediate	*icaA*, *bap*	OX, AMP, P, CX	*blaZ*	-
32		RM8	Intermediate	*icaA*, *icaB*, *icaD*	E, OX, AMP, P, CX	*blaZ*	*tst*, *PVL*
33		RM1	Intermediate	-	GEN, OX, AMP, P, CX	*mecA*, *blaZ*	-
34		RM37	Intermediate	-	OX, AMP, P, CX	*mecA*, *blaZ*	-
35		RM35	Intermediate	-	C, OX, AMP, P, CX	*mecA*, *blaZ*	-
36		RM24	Intermediate	-	E, OX, AMP, P, CX	*mecA*, *blaZ*	-
37		RM40	Intermediate	-	E, OX, AMP, P, CX	*blaZ*	-
38		RM9	Intermediate	*icaA*, *icaB*, *icaD*	E, OX, AMP, P, CX	*blaZ*	*sea*
39		RM50	Intermediate	-	E, OX, AMP, P, CX	*mecA*, *blaZ*	-
40		RM42	Intermediate	-	E, OX, AMP, P, CX	*mecA*, *blaZ*	-
41		RM51	Intermediate	-	E, GEN, OX, AMP, P, CX	*mecA*, *blaZ*	-
42		RM28	Intermediate	-	E, OX, AMP, P, CX	*blaZ*	-
43		RM15	Intermediate	-	OX, AMP, P, CX	*blaZ*	-
44		RM19	Intermediate	-	OX, AMP, P, CX	*mecA*, *blaZ*	-

45	Ready-to-eat foods	REF2(s)	Strong	-	E, OX, AMP, P, CX	*mecA, blaZ*	*sea*
46		REF20(f)	Strong	-	OX, AMP, P, CX, TE	*mecA, blaZ*	*sea, tst, PVL*
47		REF3(p)	Intermediate	-	GEN, OX, AMP, P, CX	*blaZ*	*sea, tst*
48		REF2(v)	Intermediate	-	OX, AMP, P, CX	*mecA, blaZ*	-
49		REF1(v)	Strong	*icaA, icaB, icaD, bap*	E, OX, AMP, P, CX	*mecA, blaZ*	*sea, tst, PVL*
50		REF6(s)	Strong	-	E, GEN, OX, AMP, P, CX	*mecA, blaZ*	*sea, tst, PVL*
51		REF2(f)	Intermediate	-	OX, AMP, P, CX	*mecA, blaZ*	-
52		REF6(p)	Strong	-	E, OX, AMP, P, CX	*mecA, blaZ*	*sea, tst*
53		REF1(p)	Strong	*icaA, icaB, icaD*	E, COT, OX, AMP, P, CX	*mecA, blaZ*	*sea, tst*
54		REF3(s)	Intermediate	-	OX, AMP, P, CX	*mecA, blaZ*	-
55		REF4(p)	Intermediate	-	OX, AMP, P, CX	*mecA, blaZ*	-
56		REF5(p)	Strong	*icaA, icaB, icaC, icaD, bap*	E, OX, AMP, P, CX, TE	*mecA, tetC, blaZ*	*sea, tst, PVL*
57		REF11(ff)	Intermediate	-	E, OX, AMP, P, CX	*blaZ*	-
58		REF4(ff)	Strong	-	GEN, OX, AMP, P, CX	*blaZ*	*sea*
59		REF19(ff)	Strong	-	E, OX, AMP, P, CX	*mecA, blaZ*	*sea, tst, PVL*
60		REF8(ff)	Strong	-	E, OX, AMP, P, CX	*mecA, blaZ*	*sea*
61		REF17(ff)	Intermediate	-	COT, OX, AMP, P, CX	*blaZ*	*sea, tst*
62		REF14(ff)	Strong	-	E, OX, AMP, P, CX	*blaZ*	*sea, tst, PVL*
63		REF16(ff)	Strong	-	AZM, OX, AMP, P, CX	*mecA, blaZ*	*sea, tst*
64		REF12(ff)	Intermediate	-	E, OX, AMP, P, CX, TE	*tetA, blaZ*	-
65		REF18(ff)	Strong	-	E, OX, AMP, P, CX, TE	*tetC, blaZ*	*sea, PVL*

66		REF14(f)	Strong	*icaA*, *icaB*, *icaC*, *icaD*, *bap*	GEN, OX, AMP, P, CX	*mecA*, *blaZ*	*sea*, *tst*, *PVL*
67		REF1(ff)	Intermediate	-	OX, AMP, P, CX	*mecA*, *blaZ*	-
68		REF1(f)	Strong	-	OX, AMP, P, CX	*mecA*, *blaZ*	*sea*, *tst*
69		REF20(f)	Strong	-	OX, AMP, P, CX	*mecA*, *blaZ*	*sea*, *PVL*
70		REF4(f)	Non-former	-	OX, AMP, P, CX	*blaZ*	-
71	Fish	F22	Intermediate	*icaA*, *icaB*, *icaC*, *icaD*, *bap*	E, OX, AMP, P, CX	*blaZ*	-
72		F1	Intermediate	-	OX, AMP, P, CX, TE	*tetA*, *blaZ*	*tst*
73		F19	Intermediate	-	E, OX, AMP, P, CX	*blaZ*	-
74		F4	Intermediate	-	E, OX, AMP, P, CX	*mecA*, *blaZ*	-
75		F11	Intermediate	*icaA*, *icaB*, *icaD*	OX, AMP, P, CX	*blaZ*	*sea*
76		F18	Intermediate	*icaB*, *icaD*	OX, AMP, P, CX	*blaZ*	*sea*, *tst*
77		F6	Intermediate	-	OX, AMP, P, CX	*blaZ*	-
78		F5	Intermediate	-	OX, AMP, P, CX	*blaZ*	-
79		F10	Intermediate	*icaB*, *icaD*	OX, AMP, P, CX	*mecA*, *blaZ*	*sea*
80		F2	Intermediate	-	OX, AMP, P, CX	*blaZ*	-
81		F13	Intermediate	-	OX, AMP, P, CX	*blaZ*	-
82		F16	Intermediate	-	OX, AMP, P, CX	*mecA*, *blaZ*	-
83		F9	Intermediate	-	GEN, OX, AMP, P, CX	*mecA*, *blaZ*	-
84		F3	Non-former	-	OX, AMP, P, CX	*blaZ*	-
85		F20	Intermediate	-	E, OX, AMP, P, CX	*mecA*, *blaZ*	-

86	Chicken raw meat	CM1(b)	Intermediate	-	E, OX, AMP, P, CX	*blaZ*	*sea*, *PVL*
87		CM2(t)	Intermediate	-	E, OX, AMP, P, CX	*blaZ*	-
88		CM1(t)	Strong	*icaA*, *icaB*, *icaC*, *icaD*, *bap*	OX, AMP, P, CX	*mecA*, *blaZ*	*sea*, *PVL*
89		CM3(b)	Intermediate	*icaA*, *icaB*, *icaC*, *icaD*	OX, AMP, P, CX	*mecA*, *blaZ*	*tst*
90		CM4(t)	Intermediate	*icaA*, *icaB*, *icaC*, *icaD*	OX, AMP, P, CX	*blaZ*	*sea*
91		CM4(b)	Intermediate	-	OX, AMP, P, CX	*mecA*, *blaZ*	-
92		CM3(t)	Intermediate	-	GEN, OX, AMP, P, CX	*mecA*, *blaZ*	-
93		CM5(t)	Intermediate	-	OX, AMP, P, CX	*blaZ*	-
94		CM5(b)	Strong	*icaB*, *icaD*	OX, AMP, P, CX	*mecA*, *blaZ*	*sea*, *tst*
95		CM6(b)	Intermediate	-	E, OX, AMP, P, CX	*blaZ*	-
96		CM11(b)	Intermediate	-	GEN, OX, AMP, P, CX	*mecA*, *blaZ*	-
97		CM7(t)	Intermediate	-	GEN, OX, AMP, P, CX	*mecA*, *blaZ*	-
98		CM7(b)	Intermediate	-	OX, AMP, P, CX	*mecA*, *blaZ*	-
99		CM8(b)	Intermediate	-	OX, AMP, P, CX	*mecA*, *blaZ*	-
100		CM8(t)	Intermediate	-	OX, AMP, P, CX	*mecA*, *blaZ*	-

Here, HHS= Human Hand Swab, ES= Egg Surface, RM= Raw Milk, REF= Ready-to-eat Food, v= Vegetable Fries, p= Puri, f= Fuchka, ff= French Fries, F= Fish, CM= Chicken Muscle, t= Thigh, b= Breast, C= Chloramphenicol, E= Erythromycin, COT= Co-trimoxazole, CIP= Ciprofloxacin, AZM= Azithromycin, GEN= Gentamicin, OX= Oxacillin, AMP= Ampicillin, TE= Tetracycline, P= Penicillin, CX= Cefoxitin.

Supplementary Table S2. Pearson correlation coefficients between any of two antibiotics showing to *S. aureus* isolates detected from resistance different foods and hand swab samples

		C	E	COT	CIP	AZM	GEN	TE
**C**	Pearson Correlation	1						
	Sig. (2-tailed)	-						
**E**	Pearson Correlation	-0.066	1					
	Sig. (2-tailed)	0.515	-					
**COT**	Pearson Correlation	-0.021	-0.022	1				
	Sig. (2-tailed)	0.839	0.826	-				
**CIP**	Pearson Correlation	-0.01	-0.066	-0.021	1			
	Sig. (2-tailed)	0.921	0.515	0.839	-			
**AZM**	Pearson Correlation	-0.021	-0.134	-0.042	-0.021	1		
	Sig. (2-tailed)	0.839	0.185	0.681	0.839	-		
**GEN**	Pearson Correlation	-0.035	-0.091	-0.072	-0.035	0.091	1	
	Sig. (2-tailed)	0.727	0.37	0.478	0.727	0.366	-	
**TE**	Pearson Correlation	-0.028	0.077	-0.056	-0.028	-0.056	-0.096	1
	Sig. (2-tailed)	0.785	0.447	0.58	0.785	0.58	0.34	-

Here, a *p*-value less than 0.05 was considered statistically significant, C= Chloramphenicol, E= Erythromycin, COT= Co-trimoxazole, CIP= Ciprofloxacin, AZM= Azithromycin, GEN= Gentamicin, TE= Tetracycline.
